# Editorial

**DOI:** 10.1120/jacmp.v4i2.2523

**Published:** 2003-03-01

This is the second issue from the editorial offices in snow‐deprived Minnesota. A variety of papers are presented for your edification, thanks to the patient authors and associate editors who I prod to move things along. The backlog has been reduced, and even though I am disappointed about a few papers that are taking way too long to review, I am delighted by the selection and rejection rate. John Horton will be rotating off the Editorial Board, as he is Chair of ACMP and that is a big job. I have yet to identify the proper individual (brachytherapy expertise) who will join us. Getting people to serve as AE's as well as reviewers is getting harder as each of us are becoming more accountable for our “day job” productivity. I will thank John for a yeoman job and being such a gracious person.

At RSNA I was asked by one of the brightest members of AAPM what makes JACMP different from Medical Physics. This is an important issue, as the manuscript management and online availability differences have essentially been erased. The free availability worldwide is one remaining distinction and that could be removed if Medical Physics would allow unfettered web‐based access to all its manuscripts after one year. A number of journals do this, as the income has already been earned and it gives the authors further exposure. So what remains is content. But one of my friends who has a “bear trap” mind asked, “The papers in JACMP look like those in Medical Physics?” Being the new person in the “hot seat” I decided to find the original “vision” for JACMP. Managing Editor Richard Stark provided the following “Examples of Topics Covered”:

- Reviews of emerging technology
- Clinical problems solutions
- Equipment evaluation and review articles
- New clinical techniques
- Technology integration
- Task Group recommendation implementation
- Quality assurance
- Continuous Quality Improvement
- Health Physics and radiation protection
- Changing regulatory standards
- Resource planning
- Project analysis
- Administrative issues
- Business management
- Practice accreditation
- Medical‐legal issues

So has JACMP published articles in much of these areas? The answer is some, but honestly not most. The reasons are numerous. Perhaps the “vision” is unrealistic (some topics are better as newsletter fodder). Then, again, the voluntary or solicited submissions in these areas has not been forthcoming because the vision has not been publicized, there is really nothing to say, or no one wants to waste time on these areas as they are not “academic.” As captain of the JACMP ship, I will re‐steer the rudder. The types of papers we seek will be clearly delineated, and an attempt will be made to meet this vision both in voluntary and solicited submissions. In my opinion, the continuation of JACMP depends on publishing papers that tell us how to practice Clinical Medical Physics (e.g., commissioning diodes for correct measurement of surface dose on patients), as opposed to the scientific underpinnings of the field (e.g., an investigative study of new diode construction that eliminates some of the problems of prior cnfigurations).

Other news… My request for immediate publication online at acceptance (rather than accumulation to a certain deadline) was turned down by the ACMP Board. This will be revisited in a few months. We are about ready to migrate to a new electronic manuscript handling system, which has been beta tested by Medical Physics and is now their only means of submission. The current system has some interesting quirks and Manuscript manager Sarah and I are looking to take the new system for a “spin.” Also being considered is the simplification of the journal name to Clinical Medical Physics (if available). There may be some additional reasons this is attractive, and I hope to be able to fill you in on this and related developments next time.

I really look forward to visiting you each time in this “Editorial.” It is an opportunity for me to update you, thank you for support, and ask you for ideas. Please feel free to e‐mail me (ecmc@mayo.edu) with any suggestions for how the Journal can better serve your needs both as a reader or, better still, as an author!!

Edwin C. McCullough

Editor‐in‐Chief

